# Curcumin Alleviated Dextran Sulfate Sodium-Induced Colitis by Regulating M1/M2 Macrophage Polarization and TLRs Signaling Pathway

**DOI:** 10.1155/2021/3334994

**Published:** 2021-09-16

**Authors:** Zeng-Ping Kang, Meng-Xue Wang, Tian-Tian Wu, Duan-Yong Liu, Hai-Yan Wang, Jian Long, Hai-Mei Zhao, You-Bao Zhong

**Affiliations:** ^1^Graduate School, Jiangxi University of Traditional Chinese Medicine, Nanchang 330004, Jiangxi Province, China; ^2^Formula-Pattern Research Center of Jiangxi University of Traditional Chinese Medicine, Nanchang 330004, Jiangxi Province, China; ^3^College of Traditional Chinese Medicine, Jiangxi University of Traditional Chinese Medicine, Nanchang 330004, Jiangxi Province, China; ^4^Laboratory Animal Research Center for Science and Technology, Jiangxi University of Traditional Chinese Medicine, Nanchang 330004, Jiangxi Province, China; ^5^Key Laboratory of Animal Model of TCM Syndromes of Depression, Jiangxi University of Traditional Chinese Medicine, Nanchang 330004, Jiangxi Province, China

## Abstract

Curcumin has shown good efficacy in mice with experimental colitis and in patients with ulcerative colitis, but the mechanism of action through the regulation of M1/M2 macrophage polarization has not been elaborated. The ulcerative colitis was modeled by dextran sulfate sodium; colitis mice were orally administrated with curcumin (10 mg/kg/day) or 5-ASA (300 mg/kg/day) for 14 consecutive days. After curcumin treatment, the body weight, colon weight and length, colonic weight index, and histopathological damage in colitis mice were effectively improved. The concentrations of proinflammatory cytokines IL-1*β*, IL-6, and CCL-2 in the colonic tissues of colitis mice decreased significantly, while anti-inflammatory cytokines IL-33 and IL-10 increased significantly. Importantly, macrophage activation was suppressed and M1/M2 macrophage polarization was regulated in colitis mice, and the percentage of CD11b^+^F4/80^+^ and CD11b^+^F4/80^+^TIM-1^+^ and CD11b^+^F4/80^+^iNOS^+^ decreased significantly and CD11b^+^F4/80^+^CD206^+^ and CD11b^+^F4/80^+^CD163^+^ increased significantly. Additionally, curcumin significantly downregulated CD11b^+^F4/80^+^TLR4^+^ macrophages and the protein levels of TLR2, TLR4, MyD88, NF-*κ*Bp65, p38MAPK, and AP-1 in colitis mice. Our study suggested that curcumin exerted therapeutic effects in colitis mice by regulating the balance of M1/M2 macrophage polarization and TLRs signaling pathway.

## 1. Introduction

Inflammatory bowel disease (IBD) is a chronic, relapsing, autoimmune disease of the colon and small intestine mainly comprising Crohn's disease (CD) and ulcerative colitis (UC) [1, 2]. Over the years, the incidence of IBD has been increasing year by year, and its incidence is closely related to genetic, environmental, microbial, and immune factors, among which the role of immune abnormalities has been widely concerned by scholars [3]. A growing body of evidence suggests that macrophage polarization is closely associated with the onset, activation, and remission of IBD [4–6], accompanied by a shift in macrophage phenotype. Macrophages are immune cells that can be classified into M1 and M2 types [7]. M1 macrophages are typical inflammatory cells and secreting proinflammatory cytokines IL-1*β* and IL-6, which directly lead to intestinal mucosal injury and aggravate IBD [8]. The number of M1 macrophages is significantly increased in the intestinal mucosa of DSS-induced colitis mice and active IBD patients [8]. In contrast, M2 macrophages secrete anti-inflammatory cytokines (e.g., IL-10) and are involved in tissue repair and inflammation remission to alleviate IBD [9]. Mitochondrial reactive oxygen protects the intestine from inflammation through functional M2 macrophage polarization [10]. Therefore, targeted induction of M1/M2 macrophage polarization may be a potential strategy for IBD treatment.

Curcumin is a yellow-colored bioactive polyphenolic compound extracted from the root of the turmeric plant (*Curcuma longa*) and has a wide range of physiological and pharmacological activities, including anti-inflammatory, antioxidant, anticancer, neuroprotective, and antidiabetic [11, 12]. Preclinical studies in experimental animals have shown that curcumin is effective in preventing or ameliorating intestinal inflammation in mice [13, 14]. Clinical studies have shown that curcumin is effective in combination with conventional drugs to maintain UC remission, prevent relapse, and improve clinical activity indices [15]. The potential anti-inflammatory effects of curcumin have been extensively studied in various experimental models of IBD over the last decade, including free radical scavenging, antioxidant increase, myeloperoxidase, COX-1, COX-2, LOX, TNF-*α*, IFN-*γ*, and iNOS inhibition, regulation of multiple signaling pathways, especially kinases (MAPK and ERK) and transcription factors NF-*κ*B [16, 17]. Zhong et al. also reported that curcumin effectively alleviated DSS-induced colitis, closely related to the regulation of immune memory homeostasis of T cells [18]. However, the modulatory effect of curcumin on M1/M2 macrophages in colitis mice has not been reported.

As is known, Toll-like receptors (TLRs) are a class of proteins that play a significant role in the innate immune system and are involved in inflammatory processes [19]. It is worth noting that macrophage polarization is closely associated with TLRs signaling. An elevated TLR4 expression in macrophages under action of zoledronic acid (ZA) results in higher levels of M1 macrophage polarization and lower M2 macrophage polarization both in vitro and in vivo [20]. TLR2 and TLR4 are important for activation of unprimed macrophages and that activation and effector functions induced in M1 macrophages are mainly dependent on TLR2 [21]. Furthermore, CLP-0611 inhibited the TLR4-linked NF-*κ*B and AMPK signaling pathways, polarizing M1 to M2-like macrophages and thereby ameliorating colitis [22]. Here, we investigated the effect of curcumin treatment on the development of colitis. Our results indicated that curcumin significantly decreased inflammatory responses by regulating M1/M2 macrophage polarization and TLRs signaling pathway.

## 2. Materials and Methods

### 2.1. Mice

Male BALB/c mice, aged 8–9 weeks, weighing 20–22 g, were used in this study. All mice were obtained from the Hunan Silaike Jingda Experimental Animal Co. Ltd. (Changsha, China) (Animal Certificate Number: SCXK 2019-0004). The animals were maintained under specific pathogen-free (SPF) conditions (temperature 23 ± 2°C, relative humidity 55 ± 10%, alternating 12 h light/dark cycle). All mice were fed standard food and water ad libitum and acclimatized for 3 days before the start of the experiment. The experimental protocol (permit number: JZ2019-126) was reviewed and approved by Jiangxi University of Traditional Chinese Medicine Animal Care and Use Committee and were performed in accordance with its prescribed guidelines. All mice were randomly divided into four groups: Control group, normal feeding without DSS-induced colitis; DSS group, DSS-induced colitis without drug treatment; DSS + Cur group,DSS-induced colitis treated with curcumin; DSS+5-ASA group, DSS-induced colitis treated with 5-ASA (mesalazine).

### 2.2. DSS-Induced Experimental Colitis

As described previously [23, 24], experimental colitis was first treated with a 3% (wet/vol) solution of DSS for 7 days, followed by sterile drinking water for 7 days and with 2% (wet/vol) DSS for the last 7 days. Meanwhile, the control mice received only tap water. Curcumin was supplied by GANGRUN Biotechnology (Nanjing, China) (purity >95%, High Performance Liquid Chromatography). Mesalazine (batch number: 130407) was purchased from Sunflower Pharma (Jiamusi, China). The doses of curcumin and mesalazine were based on our previous study [18], in which curcumin was added to 1.5% sodium carboxymethylcellulose. The DSS + Cur group was orally administrated with curcumin (100 mg/kg/day) for 14 consecutive days, and the DSS+5-ASA group was orally administrated with mesalazine (300 mg/kg/day), and the Control and DSS groups were given an equal volume of saline, starting from day 8. Mice were weighed at the same time each day to determine changes in body weight and monitored daily for diarrhoea, blood in the stool, humping, and hair loss [25].

### 2.3. Histological Evaluation

All mice were weighed before anesthesia, and the entire colons were rapidly collected. The total colon lengths and weights were measured [26], and the colon weight index (CWI), CWI = colon weight/body weight × 100%, was calculated [27, 28]. Distal colon tissue was taken for pathological tissue testing. The tissues were washed with phosphate-buffered saline (PBS, pH = 7.2) and fixed in 4% paraformaldehyde for 24 h at room temperature. After paraffin embedding, 4 *μ*m thick sections were cut to dehydrate by an ethanol gradient and stained with hematoxylin-eosin (H&E) (Solarbio, Beijing, China) for pathological histological analysis. Next, colon injury and inflammation were observed under a microscope (Leica, Wetzlar, Germany). Histological damage was assessed as a combined score of inflammatory cell infiltration (scores 0–3), mucosal damage (scores 0–3), crypt damage (scores 0–4), and regeneration (scores 0–4), using a previously described method [29].

### 2.4. Flow Cytometry

Briefly, spleen from mice was homogenized. The cell samples were resuspended in RPMI (Roswell Park Memorial Institute) 1640 and lysed with lysing buffer (BD Biosciences, Franklin Lakes, NJ, USA) to clear red blood cells. Then, these cells were incubated with an Fc*γ* receptor-blocking mAb (CD16/32; BioLegend, San Diego, CA, USA) for 15 minutes at 4°C. Subsequently, for surface antigen detection, the cells were shielded from light and labeled with Percp-Cy5.5 rat anti-mouse CD11b, AF647 rat anti-mouse F4/80, AF488 rat anti-mouse iNOS antibodies, PE rat anti-mouse TIM-1, PE-Cy7 rat anti-mouse TLR4, PE-Cy7 rat anti-mouse CD206, and PE rat anti-mouse CD163. All antibodies were purchased from BD Bioscience (San Jose, CA, USA). The cells were fixed and permeabilized with a Cytofix/Cytoperm Kit (BD Biosciences) prior to the standard surface and intracellular staining procedures. Finally, the single-cell suspensions were incubated for 30 min at 4°C and washed with stain buffer twice before analysis by a FACS Canto II flow cytometer (BD Biosciences, Franklin Lakes, NJ, USA). All data were analyzed with FlowJo 7.6.1 software (TreeStar, San Carlos, CA, USA). Gates were set for the quadrant markers based on negative populations and isotype controls. The numbers in the corners of the FACS dot plots represented the percentage of each cell population within that quadrant as a fraction of the total cell population.

### 2.5. Enzyme-Linked Immunosorbent Assay (ELISA)

Colon tissue (100 mg) was collected and homogenized in 1000 *μ*L RIPA (Radio Immunoprecipitation Assay) lysis buffer (Cell Signaling Technology, Danvers, MA, USA) and incubated for an hour at 4°C, followed by ultrasonic trituration and centrifugation at 10,000 rpm for 10 min to obtain colon tissue homogenate. The BCA method was used to detect the concentration of total protein and was normalised. The concentrations of cytokines were measured using mouse immunoassay kits (Thermo Fisher Scientific, Waltham, MA, USA) following the manufacturer's instruction. Enzyme-linked immunosorbent assay (ELISA) was performed to detect the levels of IL-1*β*, IL-6, IL-10, IL-33, and CCL-2 by commercial ELISA kits (Thermo Fisher Scientific, Waltham, MA, USA). 100 *μ*L of standard and sample in sequence was added to a 96-well plate and mixed thoroughly before reaction with the corresponding antibody. After terminating the reaction with 100 *μ*L of a stop solution, the optical density (OD) values of these cytokines in each sample were detected using a microplate reader (Thermo, Varioskan Flash, MA, USA).

### 2.6. Western Blot Analysis

Colon tissue protein samples were prepared in accordance with the protein samples in 2.5. Equal weight of protein per sample was separated using SDS-PAGE gels and transferred to PVDF membranes. These membranes were blocked with 3% BSA for 1 h at room temperature and then incubated with the indicated primary antibody overnight at 4°C, including anti-GAPDH (1 : 1000), AP-1 (1 : 1000), MyD88 (1 : 600), p38MAPK (1 : 600), NF-*κ*Bp65 (1 : 1000), TLR2 (1 : 500), and TLR4 (1 : 500) antibodies. The HRP-coupled secondary antibody was then added. Protein detection was performed using the ECL substrate (Thermo, Rockford, IL, USA) before exposure, and photos were taken using the Highly Sensitive Chemiluminescence Imaging system (UVP ChemStudio 515; Analytik Jena, Jena, Germany). The images were quantified using Image-Pro Plus 6.0 software (Media Cybernetic, Bethesda, MD, USA).

### 2.7. Statistics

All data were presented as mean ± standard error of the mean (SEM). Statistical analysis was performed by one-way analysis of variance (ANOVA) followed by the least-significant difference (LSD) test to compare the differences between every two groups in GraphPad Prism 7.0 software (San Diego, CA, USA). *P* < 0.05 was considered as significant difference.

## 3. Results

### 3.1. Curcumin Alleviated DSS-Induced Colitis

**Figure 1:**
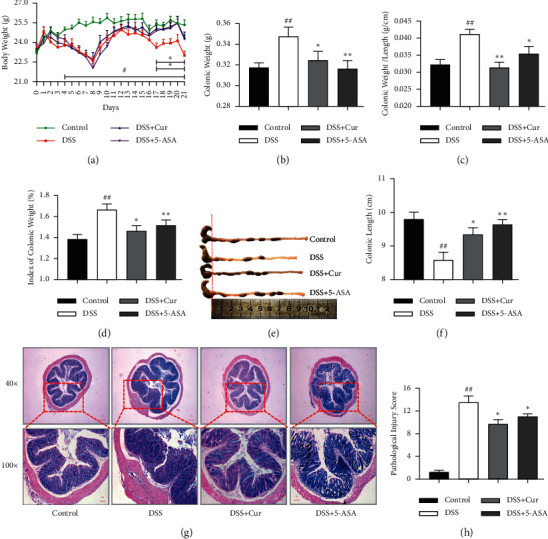
Curcumin relieved DSS-induced colitis in mice. (a) The body weight from day 0 to day 21. (b) The colonic weight. (c) Colonic weight/length (g/cm). (d) Index of colonic weight. (e) Changes in colonic length by the naked eye. (f) Colonic length. (g) HE staining. (h) Histopathological score. Data are expressed as mean ± SEM (*n* = 8). ^#^*P* < 0.05 and ^##^*P* < 0.01 versus the Control group. ^*∗*^*P* < 0.05 and ^*∗∗*^*P* < 0.01 versus the DSS group.

In the present study, the body weight (Figure 1(a)) of DSS-induced colitis mice decreased significantly from day 4 to day 21 of the experiment, colonic weight (Figure 1(b)), colonic weight/length (Figure 1(c)) and index of colonic weight (Figure 1(d)) also increased significantly, and colonic length decreased significantly (Figures 1(e) and 1(f)). Meanwhile, colonic histological disorders, loss of intestinal crypts, mucosal ulceration, lymphocytic infiltration, and edema (Figure 1(g)), and significant upregulation of pathological injury scores (Figure 1(h)) were observed in colitis mice. These findings indicated that the chronic colitis was successfully modeled in this study.

After treatment with curcumin and mesalazine, effective reversal of the significant changes in body weight, index of colon weight, colon weight, and colon length and histopathology in colitis mice was achieved. And colonic weight (Figure 1(b)), colonic weight/length (Figure 1(c)), and index of colonic weight (Figure 1(d)) were significantly lower in the DSS + Cur and DSS+5-ASA groups than in the DSS group, and colonic length (Figures 1(e) and 1(f)) was significantly higher in the DSS + Cur group than in the DSS group. In addition, the ulcer formation and inflammatory infiltration (Figure 1(g)) were significantly improved, and the pathological damage scores (Figure 1(h)) were significantly downregulated in colitis mice. Therefore, curcumin can effectively alleviate DSS-induced colonic injury.

### 3.2. Curcumin Regulated the Expression of Inflammatory Cytokines in Colon Tissues

**Figure 2:**
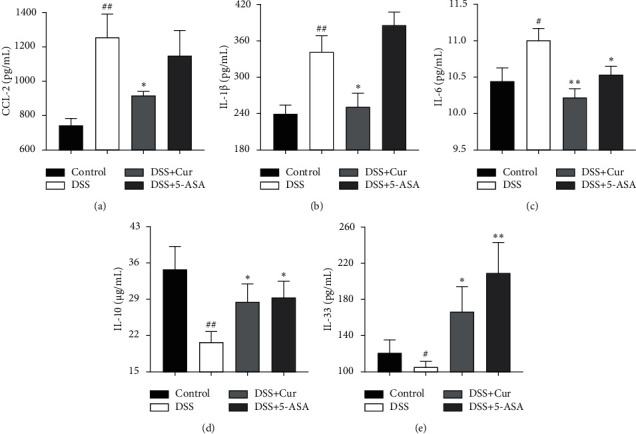
Curcumin regulated the expression levels of inflammatory cytokines in colitis mice. The levels of proinflammatory cytokines (a) CCL-2, (b) IL-1*β*, and (c) IL-6 and anti-inflammatory cytokines (d) IL-10 and (e) IL-33 were measured by ELISA. Data are expressed as mean ± SEM (*n* = 8). ^#^*P* < 0.05 and ^##^*P* < 0.01 compared with the Control group. ^*∗*^*P* < 0.05 and ^*∗∗*^*P* < 0.01 versus the DSS group.

In this study, the levels of the proinflammatory cytokines CCL-2 (Figure 2(a)), IL-1*β* (Figure 2(b)), and IL-6 (Figure 2(c)) were remarkably increased in the DSS group than in the Control group (Figures 2(a), 2(b), and 2(c)), and the anti-inflammatory cytokines IL-33 (Figure 2(d)) and IL-10 (Figure 2(e)) in the DSS group were dramatically decreased. Importantly, the concentrations of CCL-2 (Figure 2(a)), IL-1*β* (Figure 2(b)), and IL-6 (Figure 2(c)) were significantly downregulated after DSS-induced colitis mice were treated with curcumin; IL-33 (Figure 2(d)) and IL-10 (Figure 2(e)) were significantly upregulated. Altogether, curcumin effectively alleviated DSS-induced colonic mucosal damage by modulating the release of inflammatory cytokines.

### 3.3. Curcumin Inhibited the Activation of Macrophages in Colitis Mice

**Figure 3:**
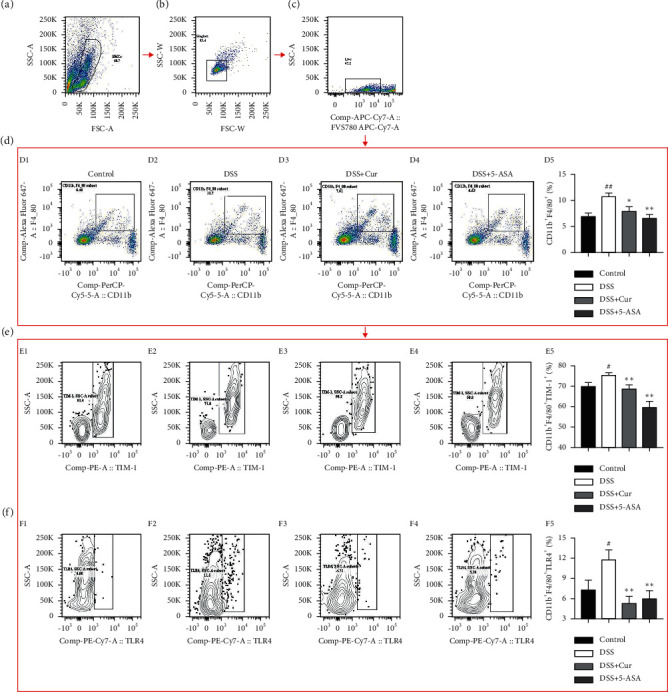
Curcumin inhibited the activation of macrophages in colitis mice. (a) Splenic mononuclear cells (SMCs). (b) Singlets. (c) Live cells. (d) Analysis of CD11b^+^F4/80^+^ cells from these four groups. (e) Analysis of macrophage-associated activation molecules on CD11b^+^F4/80^+^TIM-1^+^cells from these four groups. (f) Analysis of TLR4 signal molecules on CD11b^+^F4/80^+^cells from these four groups. D1, E1, F1: Control group; D2, E2, F2: DSS group; D3, E3, F3: DSS + Cur group; D4, E4, F4: DSS+5-ASA group; D5: statistical analysis of CD11b^+^F4/80^+^cells percentage; E5: statistical analysis of CD11b^+^F4/80^+^TIM-1^+^ cells percentage; F5: statistical analysis of CD11b^+^F4/80^+^TLR4^+^ cells percentage. Data are expressed as mean ± SEM (*n* = 8). ^#^*P* < 0.05 and ^##^*P* < 0.01 versus the Control group. ^*∗*^*P* < 0.05 and ^*∗∗*^*P* < 0.01 versus the DSS group.

In our study, the percentage of CD11b^+^F4/80^+^ (Figure 3(d)) macrophages was significantly increased in the spleen of colitis mice, whereas the percentage of CD11b^+^F4/80^+^ (Figure 3(d)) macrophages was significantly decreased after curcumin administration treatment. Activation of macrophages results in the expression of TIM-1, the expression level of which is positively correlated with the status of macrophage activation [30]. We found a significant increase in the percentage of CD11b^+^F4/80^+^TIM-1^+^ macrophages (Figure 3(e)) in colitis mice, whereas curcumin administration treatment resulted in a significant decrease in the percentage of CD11b^+^F4/80^+^TIM-1^+^ macrophages (Figure 3(e)). Interestingly, the percentage of CD11b^+^F4/80^+^TLR4^+^ macrophages (Figure 3(f)) in colitis mice increased significantly, whereas curcumin administration treatment resulted in a significant decrease in the percentage of CD11b^+^F4/80^+^TLR4^+^ macrophages (Figure 3(f)). These studies suggested that curcumin inhibited macrophage activation and thus interfered with the process of experimental colitis, possibly in close association with TLR4 signaling pathway.

### 3.4. Curcumin Suppressed the Activation of TLRs Signaling Pathway in Colitis Mice

**Figure 4:**
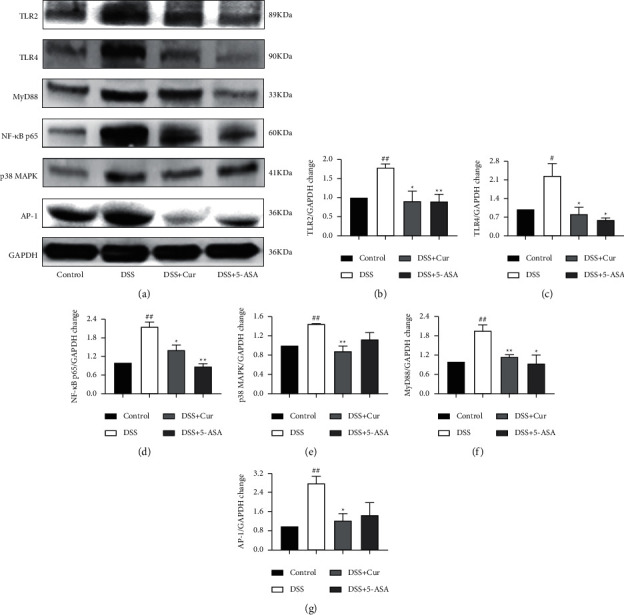
Curcumin inhibited the activation of TLRs signaling pathway in colitis mice. After administration of curcumin, the expression levels of TLRs signaling related proteins in colon were detected by western blotting to determine whether the TLRs signaling pathway was activated. (a) Western blotting of major proteins in the TLRs/MyD88 signaling pathway, such as NF-*κ*Bp65, p38MAPK, and AP-1. (b) TLR2/GAPDH change. (c) TLR4/GAPDH change. (d) NF-*κ*Bp65/GAPDH change. (e) p38MAPK/GAPDH change. (f) MyD88/GAPDH change. (g) AP-1/GAPDH change. Data are expressed as mean ± SEM (*n* = 8). ^#^*P* < 0.05 and ^##^*P* < 0.01 versus the Control group. ^*∗*^*P* < 0.05 and ^*∗∗*^*P* < 0.01 versus the DSS group.

Naturally, the effects of curcumin on the regulation of TLRs signaling pathways in colitis mice were further investigated by Western blot analysis, including TLRs signaling molecules TLR2 and TLR4 and the downstream proteins MyD88, NF-*κ*Bp65, p38MAPK, and AP-1. In our study, the expression levels of TLR2 and TLR4 and those of the downstream proteins NF-*κ*Bp65, p38MAPK, and AP-1 (Figure 4) were significantly higher in DSS group than in the Control group. These results indicated that the TLRs signaling pathway was activated in DSS-induced colitis mice. After curcumin treatment, the protein levels of TLR2, TLR4, MyD88, NF-*κ*Bp65, p38MAPK, and AP-1 (Figure 4) in the colonic tissues of colitis mice were significantly reduced. Thus, these data suggested that curcumin inhibited the activation of TLRs signaling pathway in colitis mice.

### 3.5. Curcumin Regulated the Polarization Balance of M1/M2 Macrophage in Colitis Mice

**Figure 5:**
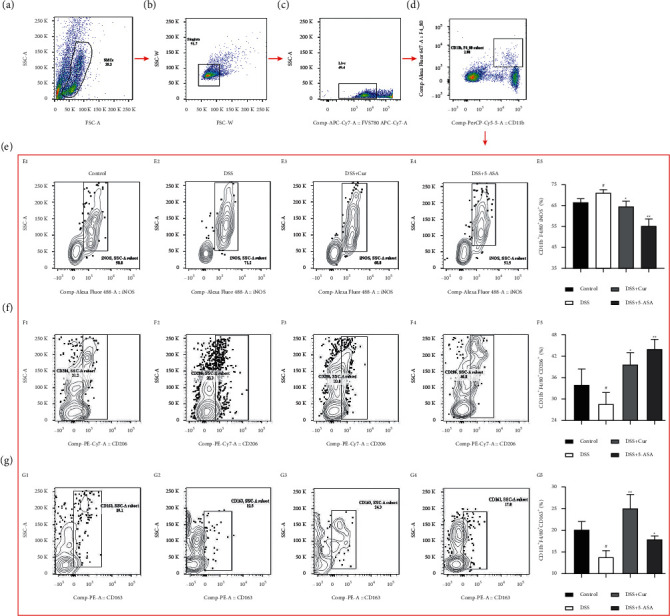
Curcumin regulated the polarization balance of M1/M2 macrophages in colitis mice. (a) Splenic mononuclear cells (SMCs). (b) Singlets. (c) Live cells. (d) CD11b^+^F4/80^+^ cells. (e) Analysis of M1 macrophage-associated molecule iNOS on CD11b^+^F4/80^+^cells from these four groups. (f, g) Analysis of M2 macrophage-associated molecule CD206 and CD163 on CD11b^+^F4/80^+^cells from these four groups. E1, F1, G1: Control group; E2, F2, G2: DSS group; E3, F3, G3: DSS + Cur group; E4, F4, G4: DSS+5-ASA group; E5: statistical analysis of CD11b^+^F4/80^+^iNOS^+^ cells percentage; F5: statistical analysis of CD11b^+^F4/80^+^CD206^+^ cells percentage; G5: statistical analysis of CD11b^+^F4/80^+^CD163^+^ cells percentage. Data are expressed as mean ± SEM (*n* = 8). ^#^*P* < 0.05 and ^##^*P* < 0.01 versus the Control group. ^*∗*^*P* < 0.05 and ^*∗∗*^*P* < 0.01 versus the DSS group.

Disruption of colonic homeostasis caused by aberrant M1/M2 macrophage polarization and dysbiosis contributes to IBD pathogenesis [31]. Secretion of iNOS by proinflammatory M1 macrophages is typical [32], and the surface markers for anti-inflammatory M2 macrophages are CD206 and CD163 [33]. In this study, the percentage of M1 macrophage CD11b^+^F4/80^+^iNOS^+^ (Figure 5(e)) was significantly increased in DSS-induced colitis mice, and those of M2 macrophage CD11b^+^F4/80^+^CD206^+^ (Figure 5(f)) and CD11b^+^F4/80^+^CD163^+^ (Figure 5(g)) cells were significantly downregulated. It suggested that DSS-induced colitis disrupted the differentiation balance of M1/M2 macrophage. After administration of Cur, the percentage of CD11b^+^F4/80^+^iNOS^+^ (Figure 5(e)) macrophages in colitis mice was significantly downregulated, and those of M2 macrophage CD11b^+^F4/80^+^CD206^+^ (Figure 5(f)) and CD11b^+^F4/80^+^CD163^+^ (Figure 5(g)) macrophages were significantly upregulated. These results suggested that curcumin effectively regulated the balance of M1/M2 macrophage polarization in colitis mice.

## 4. Discussion

Mesalazine, also known as 5-aminosalicylic acid (5-ASA), is a first-line treatment for many patients with IBD [34]. 5-ASA has a wide range of anti-inflammatory effects, including blockade of nuclear factor-*κ*B (NF-*κ*B) signaling, downregulation of proinflammatory cytokines, inhibition of cyclooxygenase-2 (COX-2), eicosanoids, prostaglandin E2 (PGE2), and leukotriene B4 (LTB4), and reduction of oxidative stress [35]. 5-ASA has shown good efficacy in a variety of experimental animal models [36, 37] and is often used in the development of new drugs. In this study, curcumin and 5-ASA effectively prevented the changes of the body weight, colonic weight and length, colonic weight index, and the histopathological damage in colitis mice. This implies that the efficacy of curcumin in DSS-induced colitis mice is similar to that of 5-ASA.

Next, we further investigated the action mechanism of curcumin in alleviating colitis. Macrophages polarized to different phenotypes critically contribute to colitis development by coordinating inflammatory and anti-inflammatory processes [38]. In this study, abnormal macrophage activation and M1/M2 macrophage polarization imbalance were found in DSS-induced colitis mice. Recently, targeting the balance between proinflammatory M1 and anti-inflammatory M2 macrophage phenotypes may be a new therapeutic approach for colitis [39]. We found that curcumin inhibited macrophage activation and regulated M1/M2 macrophage polarization in colitis mice. Previous studies have shown that the M1 macrophages are first activated when the intestinal mucosal barrier is disrupted and a continuous stimulus by an antigen is exerted, releasing TNF-*α*, IL-1, IL-6, reactive nitrogen, and superoxide intermediates in a response to counteract the stimulation, while the M2 macrophage effector functions are suppressed and the levels of IL-10 and IL-33 downregulated [33, 40]. In addition, we found that curcumin promoted the release of the anti-inflammatory cytokines IL-10 and IL-33 in colitis mice and inhibited the secretion of the proinflammatory cytokines CCL-2, IL-1*β*, and IL-6. Interestingly, the change trends of proinflammatory cytokines and anti-inflammatory cytokines in curcumin-regulated colitis mice were consistent with the trends of changes in M1 and M2 macrophages, respectively. Our present results provide new insights onto the therapeutic effect of curcumin in ameliorating DSS-induced experimental colitis by regulating M1/M2 macrophage polarization and their effector functions.

TLRs signaling pathway plays an important role in macrophage activation and is the most potent activator of the inflammatory response [41, 42]. IBD is an inflammatory disorder, characterized by abnormally increased expression of the TLR2 and TLR4 in the colon and increased proinflammatory cytokine production by macrophages [43, 44]. In addition, TLRs signaling is an important pathway of M1 macrophage activation in the DSS-induced inflammatory response [42]. MyD88 is an adaptor protein required for the induction of most TLRs into proinflammatory cytokines. The activation of the TLRs/MyD88 signaling pathway in colitis has been reported to lead to the secretion of large amounts of cytokines by macrophages [45]. Triggering TLR ligands sequentially activate MyD88, p38MAPK, AP-1 [46], and NF-*κ*Bp65, which is involved in the transcriptional activation of several inflammatory genes [47]. Our present results showed that the percentage of CD11b^+^F4/80^+^TLR4^+^ macrophages of colitis mice increased significantly and the protein expression levels of TLR2, TLR4, NF-*κ*Bp65, p38MAPK, MyD88, and AP-1 in mouse colonic tissues were increased significantly, suggesting that TLRs signaling-mediated macrophage activation is involved in the pathogenesis of colitis. Notably, curcumin significantly decreased the percentage of CD11b^+^F4/80^+^TLR4^+^ macrophages and inhibited the expression of TLRs/MyD88 signaling molecules and their downstream proteins in colitis mice. These results suggest that curcumin may interfere with the polarization and effector functions of macrophages through the inhibition of the TLRs signaling pathway, thereby inhibiting the release of proinflammatory cytokines and ameliorating experimental colitis.

Currently, it is unquestionable that macrophages express TLR2 and TLR4 [48]. Our study showed that curcumin inhibited the TLR signaling pathway and M1/M2 macrophage polarization in DSS-induced colitis mice, and found that the M1/M2 macrophage phenotypic switch was closely associated with TLR signaling. This has laid the foundation for our later studies on the key targets of curcumin.

## Data Availability

The data presented in this study are available from the corresponding author on reasonable request.

## References

[1] Hibi T., Imai Y., Senoo A., Ohta K., Ukyo Y. (2017). Efficacy and safety of golimumab 52-week maintenance therapy in Japanese patients with moderate to severely active ulcerative colitis: a phase 3, double-blind, randomized, placebo-controlled study-(PURSUIT-J study). *Journal of Gastroenterology*.

[2] Zhang Y.-Z., Li Y. Y. (2014). Inflammatory bowel disease: pathogenesis. *World Journal of Gastroenterology*.

[3] Na Y. R., Stakenborg M., Seok S. H., Matteoli G. (2019). Macrophages in intestinal inflammation and resolution: a potential therapeutic target in IBD. *Nature Reviews Gastroenterology & Hepatology*.

[4] Lissner D., Schumann M., Batra A. (2015). Monocyte and M1 macrophage-induced barrier defect contributes to chronic intestinal inflammation in IBD. *Inflammatory Bowel Diseases*.

[5] Orecchioni M., Ghosheh Y., Pramod A. B., Ley K. (2019). Macrophage polarization: different gene signatures in M1(LPS+) vs. Classically and M2(LPS-) vs. Alternatively activated macrophages. *Frontiers in Immunology*.

[6] Park S.-W., Kim T. J., Lee J. Y. (2019). Comorbid immune-mediated diseases in inflammatory bowel disease: a nation-wide population-based study. *Alimentary Pharmacology & Therapeutics*.

[7] Wu Y., Wu B., Zhang Z. (2020). Heme protects intestinal mucosal barrier in DSS‐induced colitis through regulating macrophage polarization in both HO‐1‐dependent and HO‐1‐independent way. *The FASEB Journal*.

[8] Lin Y., Yang X., Yue W. (2014). Chemerin aggravates DSS-induced colitis by suppressing M2 macrophage polarization. *Cellular & Molecular Immunology*.

[9] Loftus E. V. (2004). Clinical epidemiology of inflammatory bowel disease: incidence, prevalence, and environmental influences. *Gastroenterology*.

[10] Formentini L., Santacatterina F., Núñez de Arenas C. (2017). Mitochondrial ROS production protects the intestine from inflammation through functional M2 macrophage polarization. *Cell Reports*.

[11] Yue W., Liu Y., Liu Y. (2019). Curcumin ameliorates dextran sulfate sodium-induced colitis in mice via regulation of autophagy and intestinal immunity. *Turkish Journal of Gastroenterology*.

[12] Simadibrata M., Halimkesuma C. C., Suwita B. M. (2017). Efficacy of curcumin as adjuvant therapy to induce or maintain remission in ulcerative colitis patients: an evidence-based clinical review. *Acta Medica Indonesiana*.

[13] Kunnumakkara A. B., Bordoloi D., Padmavathi G. (2017). Curcumin, the golden nutraceutical: multitargeting for multiple chronic diseases. *British Journal of Pharmacology*.

[14] Nabavi S., Thiagarajan R., Rastrelli L. (2015). Curcumin: a natural product for diabetes and its complications. *Current Topics in Medicinal Chemistry*.

[15] Sadeghi N., Mansoori A., Shayesteh A., Hashemi S. J. (2020). The effect of curcumin supplementation on clinical outcomes and inflammatory markers in patients with ulcerative colitis. *Phytotherapy Research*.

[16] Wang Q., Ye C., Sun S. (2019). Curcumin attenuates collagen-induced rat arthritis via anti-inflammatory and apoptotic effects. *International Immunopharmacology*.

[17] Giordano A., Tommonaro G. (2019). Curcumin and cancer. *Nutrients*.

[18] Zhong Y. B., Kang Z. P., Zhou B. G. (2020). Curcumin regulated the homeostasis of memory T cell and ameliorated dextran sulfate sodium-induced experimental colitis. *Frontiers in Pharmacology*.

[19] Kordjazy N., Haj-Mirzaian A., Haj-Mirzaian A. (2018). Role of toll-like receptors in inflammatory bowel disease. *Pharmacological Research*.

[20] Zhu W., Xu R., Du J. (2019). Zoledronic acid promotes TLR‐4‐mediated M1 macrophage polarization in bisphosphonate‐related osteonecrosis of the jaw. *The FASEB Journal*.

[21] Holden J. A., O’Brien-Simpson N. M., Lenzo J. C., Orth R. K. H., Mansell A., Reynolds E. C. (2017). Porphyromonas gulae activates unprimed and gamma interferon-primed macrophages via the pattern recognition receptors toll-like receptor 2 (TLR2), TLR4, and NOD2. *Infection and Immunity*.

[22] Jang S.-E., Han M. J., Kim S.-Y., Kim D.-H. (2014). Lactobacillus plantarum CLP-0611 ameliorates colitis in mice by polarizing M1 to M2-like macrophages. *International Immunopharmacology*.

[23] Ginzel M., Feng X., Kuebler J. F. (2017). Dextran sodium sulfate (DSS) induces necrotizing enterocolitis-like lesions in neonatal mice. *PLoS One*.

[24] Jerkic M., Peter M., Ardelean D., Fine M., Konerding M. A., Letarte M. (2010). Dextran sulfate sodium leads to chronic colitis and pathological angiogenesis in Endoglin heterozygous mice. *Inflammatory Bowel Diseases*.

[25] Sann H., Erichsen J. v., Hessmann M., Pahl A., Hoffmeyer A. (2013). Efficacy of drugs used in the treatment of IBD and combinations thereof in acute DSS-induced colitis in mice. *Life Sciences*.

[26] Chassaing B., Aitken J. D., Malleshappa M., Vijay-Kumar M. (2014). Dextran sulfate sodium (DSS)-induced colitis in mice. *Current Protocols in Immunology*.

[27] Zuo T., Yue Y., Wang X., Li H., Yan S. (2020). Luteolin relieved DSS-induced colitis in mice via HMGB1-TLR-NF-*κ*B signaling pathway. *Inflammation*.

[28] Tozaki H., Odoriba T., Okada N. (2002). Chitosan capsules for colon-specific drug delivery: enhanced localization of 5-aminosalicylic acid in the large intestine accelerates healing of TNBS-induced colitis in rats. *Journal of Controlled Release*.

[29] Dieleman L. A., Palmen M. J., Akol H. (1998). Chronic experimental colitis induced by dextran sulphate sodium (DSS) is characterized by Th1 and Th2 cytokines. *Clinical & Experimental Immunology*.

[30] Hein R. M., Woods M. L. (2007). TIM-1 regulates macrophage cytokine production and B7 family member expression. *Immunology Letters*.

[31] Horuluoglu B. H., Kayraklioglu N., Tross D., Klinman D. (2020). PAM3 protects against DSS-induced colitis by altering the M2:M1 ratio. *Scientific Reports*.

[32] Klug F., Prakash H., Huber P. E. (2013). Low-dose irradiation programs macrophage differentiation to an iNOS+/M1 phenotype that orchestrates effective T cell immunotherapy. *Cancer Cell*.

[33] Jiang M., Liu X., Zhang D. (2018). Celastrol treatment protects against acute ischemic stroke-induced brain injury by promoting an IL-33/ST2 axis-mediated microglia/macrophage M2 polarization. *Journal of Neuroinflammation*.

[34] Sehgal P., Colombel J.-F., Aboubakr A., Narula N. (2018). Systematic review: safety of mesalazine in ulcerative colitis. *Alimentary Pharmacology & Therapeutics*.

[35] Yorulmaz E., Yorulmaz H., Gokmen E. S. (2019). Therapeutic effectiveness of rectally administered fish oil and mesalazine in trinitrobenzenesulfonic acid-induced colitis. *Biomedicine & Pharmacotherapy*.

[36] Vishwakarma N., Ganeshpurkar A., Pandey V., Dubey N., Bansal D. (2015). Mesalazine-probiotics beads for acetic acid experimental colitis: formulation and characterization of a promising new therapeutic strategy for ulcerative colitis. *Drug Delivery*.

[37] Ribaldone D. G., Vernero M., Caviglia G. P. (2021). Targeting IL‐10, ZO‐1 gene expression and IL‐6/STAT‐3 trans‐signalling by a combination of atorvastatin and mesalazine to enhance anti‐inflammatory effects and attenuate progression of oxazolone‐induced colitis. *Fundamental & Clinical Pharmacology*.

[38] Zhuang H., Lv Q., Zhong C. (2021). Tiliroside ameliorates ulcerative colitis by restoring the M1/M2 macrophage balance via the HIF-1*α*/glycolysis pathway. *Frontiers in Immunology*.

[39] Castro-Dopico T., Fleming A., Dennison T. W. (2020). GM-CSF calibrates macrophage defense and wound healing programs during intestinal infection and inflammation. *Cell Reports*.

[40] Gomes V. J., Nunes P. R., Matias M. L. (2020). Silibinin induces in vitro M2-like phenotype polarization in monocytes from preeclamptic women. *International Immunopharmacology*.

[41] Quero L., Hanser E., Manigold T., Tiaden A. N., Kyburz D. (2017). TLR2 stimulation impairs anti-inflammatory activity of M2-like macrophages, generating a chimeric M1/M2 phenotype. *Arthritis Research & Therapy*.

[42] Komai K., Shichita T., Ito M., Kanamori M., Chikuma S., Yoshimura A. (2017). Role of scavenger receptors as damage-associated molecular pattern receptors in Toll-like receptor activation. *International Immunology*.

[43] Shi Y.-J., Gong H.-F., Zhao Q.-Q., Liu X.-S., Liu C., Wang H. (2019). Critical role of toll-like receptor 4 (TLR4) in dextran sulfate sodium (DSS)-Induced intestinal injury and repair. *Toxicology Letters*.

[44] Dong L., Li J., Liu Y., Yue W., Luo X. (2012). Toll-like receptor 2 monoclonal antibody or/and Toll-like receptor 4 monoclonal antibody increase counts of Lactobacilli and Bifidobacteria in dextran sulfate sodium-induced colitis in mice. *Journal of Gastroenterology and Hepatology*.

[45] Han C., Xu J., Liu C. (2018). Modulation of TLR2 and TLR4 in macrophages following Trichinella spiralis infection. *Helminthologia*.

[46] Wang B., Wu Y., Liu R. (2020). Lactobacillus rhamnosus GG promotes M1 polarization in murine bone marrow-derived macrophages by activating TLR2/MyD88/MAPK signaling pathway. *Animal science journal = Nihon chikusan Gakkaiho*.

[47] Hu X., Chen J., Wang L., Ivashkiv L. B. (2007). Crosstalk among Jak-STAT, Toll-like receptor, and ITAM-dependent pathways in macrophage activation. *Journal of Leukocyte Biology*.

[48] Ma K. L., Han Z. J., Pan M. (2020). Therapeutic effect of cinnamaldehyde on ulcerative colitis in mice induced by dextran sulfate sodium with Candida albicans colonization and its effect on dectin-1/TLRs/NF-*κ*B signaling pathway. *Zhongguo Zhongyao Zazhi*.

